# Differences in acute outcomes of suicide patients by psychiatric disorder: Retrospective observational study

**DOI:** 10.1097/MD.0000000000035065

**Published:** 2023-09-22

**Authors:** Takumi Tsuchida, Masaki Takahashi, Asumi Mizugaki, Hisashi Narita, Takeshi Wada

**Affiliations:** a Division of Acute and Critical Care Medicine, Department of Anesthesiology and Critical Care Medicine, Hokkaido University Faculty of Medicine, Sapporo, Japan; b Department of Psychiatry, Hokkaido University Faculty of Medicine, Sapporo, Japan.

**Keywords:** mental disease, mortality, outcome, psychiatric disorders, self-harm, suicide, suicide risk

## Abstract

Suicide is a social problem with significant economic losses, the victims of which are mainly from the productive population. There are numerous reports on the assessment of suicide risk, but most focus on long-term management. Therefore, factors influencing the severity of physical impairments in the acute phase and the prognosis of suicidal patients have not been sufficiently investigated. This is a single-center retrospective observational study. We collected data on suicidal patients admitted to our emergency department. The effect of age, gender, psychiatric history, method of suicide, alcohol consumption, and hospital admission on the outcome of suicide was assessed. Outcomes were assessed using the hospital mortality scale and the cerebral performance category scale for in-hospital mortality within 28 days. Methods of suicide with a high mortality rate (hanging, jumping, carbon monoxide poisoning, and burns) were defined as lethal methods. A detailed risk assessment of outcomes was performed for patients with schizophrenia, mood disorders, and somatoform disorders. We identified 340 suicide patients from computerized medical records and analyzed 322 records without missing data. The non-survivor group predominantly comprised older adults, men, and patients without a history of psychiatric treatment. Contrastingly, more patients drank alcohol before suicide in the survivor group. In the subgroup analysis, patients with schizophrenia had unfavorable neurological outcomes. Patients with mood disorders had worse in-hospital mortality than other psychiatric patients, as did patients who chose the lethal method. By disease, patients with stress-related and somatoform disorders tended to have higher survival rates, although their psychiatric hospitalization rates were lower. Conversely, patients with mood disorders had a higher rate of hospital visits but a lower survival rate. The results suggest that usual outpatient treatment alone may not be sufficient to reduce suicide mortality in patients with mood disorders who are considered to be at high risk of suicide.

## 1. Introduction

Every year, approximately 700,000 people worldwide take their own lives and many more attempt suicide. People of all ages commit suicide; it was found to be the fourth leading cause of death among 15- to 29-year-olds worldwide in 2019.^[1]^ Suicide is a serious problem worldwide the importance of this issue has increased in recent years owing to the COVID-19 pandemic.^[2–6]^ Japan has a particularly high suicide rate among working-age men,^[7]^ and suicide remains 1 of top 10 causes of death in Japan.^[8]^

As suicide is a serious problem, various studies have been conducted on its prevention. However, relatively few studies have been conducted on patients after suicide, and there are limited reports on the physical severity of the immediate post-suicide period.^[9–13]^ Furthermore, these studies do not provide sufficient information on psychiatric history^[12,13]^ or include only suicide survivors.^[9–11]^ Therefore, factors affecting survival and neurological outcomes in suicide patients have not been adequately investigated.

In this study, we hypothesized that the means of suicide chosen and the strength of the will to complete suicide, that is, the degree of injury, would differ depending on the psychiatric disorder. We also aimed to identify the factors associated with survival rate, functional outcome, and physical severity of suicide in all suicidal patients admitted to the emergency department.

## 2. Materials and methods

### 2.1. Patient selection and data collection

We identified 340 out of 11,233 patients who were admitted to Hokkaido University Hospital for suicide between June 2010 and May 2021. This patient information was extracted from the computerized medical records that contain information on all patients who have been transported to this hospital. Patients were identified based on the certainty that suicide was the cause of the emergency department visit. The suicide situation, psychiatric history, and medication history of the selected patients were identified from the database, along with their basic information. In Japan, patient information is recorded in detail by the emergency medical services (EMS) according to a prescribed form. Information on the circumstances of the suicide scene, alcohol consumption, medications, and medical history were obtained in detail from the EMS and patient relatives. Patient information was obtained from the descriptions on the prescribed forms obtained from the EMS and from the medical records, which include a detailed interview with the patient attending physician. Psychiatric history was obtained according to diagnoses by a nearby psychiatrist or psychiatric specialist at Hokkaido University Hospital. Psychiatric disorders were identified and extracted using the International Statistical Classification of Diseases and Related Health Problems, Tenth Revision (ICD-10).

### 2.2. Setting

Hokkaido University Hospital has 922 beds and accepts more than 1000 patients with the most severe conditions each year. There are 16 emergency doctors and approximately 30 nurses in the emergency department, with 2 to 3 doctors and 2 to 3 nurses to respond to 1 transported emergency patient. Hokkaido University Hospital is a tertiary care center in Sapporo City, Japan, which covers an area of 1121 km^[2]^ with a population of approximately 2.0 million. There are 5 tertiary care centers in Sapporo, and all critically injured patients are transported to the nearest tertiary care center. EMS personnel are legally prohibited from terminating resuscitation at the scene, and all patients are transported to the hospital unless death is inevitable. All patients brought to the facility receive standard acute care. Patients receiving liaison interventions are diagnosed through conferences with several psychiatrists, including a board-certified psychiatrist. Suicide by firearm is rare in Japan, as the possession of firearms is prohibited by law. Autopsies and on-site inspections are performed on all patients whose deaths are externally caused. This study included patients whose cause of death was determined to be suicide after a detailed investigation by the police.

### 2.3. Outcomes and definitions

Outcomes were assessed using the hospital mortality and cerebral performance category (CPC) scale at 28 hospital days. We defined CPCs 1 and 2 as favorable neurological outcomes and CPCs 3 to 5 as unfavorable neurological outcomes.

The definition of psychiatric disorders using the ICD-10 classification in this study is as follows: F0: organic, including symptomatic, mental disorders; F1: mental and behavioral disorders owing to psychoactive substance use; F2: schizophrenia, schizotypal, and delusional disorders; F3: mood disorders; F4: neurotic, stress-related, and somatoform disorders; F5: behavioral syndromes associated with physiological disturbances and physical factors; F6: disorders of adult personality and behavior; F7: mental retardation; and F8: pervasive developmental disorders. The analysis was conducted using the broad F categories as classifying the diseases in more detail could have resulted in an insufficient number of cases for meaningful analysis.

### 2.4. Study design

This is a single-center, retrospective study using data from patients previously transferred to Hokkaido University Hospital. To address potential sources of bias, data collection, outcome measurement, and statistical analysis were performed by 3 independent investigators.

### 2.5. Ethics

The study protocol was approved by our institutional review board (approval number: 021-0006, approval date: June 3, 2021), and the requirement for informed consent was waived owing to the retrospective design. Information about the conduct of the study, including the purpose of the study, was published on the website. Patients and their relatives were given the opportunity to refuse to participate in the study.

### 2.6. Statistical analysis

Patients were divided into the survivor and non-survivor groups, and the effect of each variable on outcome was assessed. We defined suicide methods with high mortality rates (hanging, jumping, carbon monoxide [CO] intoxication, and burning) as lethal methods based on previous reports^[14,15]^ and performed a subgroup analysis of patients with F2, F3, and F4 classifications. All diagnosed patients without missing data were analyzed for F2 versus non-F2, F3 versus non-F3, and F4 versus non-F4 patients by propensity score matching for age, gender, and lethal method. We also performed a propensity score matching for patients who chose lethal methods, with age and gender as covariates. Additional analyses were performed for F2 versus non-F2 patients, F3 versus non-F3 patients, and F4 versus non-F4 patients. In addition to the disease-specific analysis, patients were divided into 2 groups according to whether they had drunk alcohol shortly before the suicide. The characteristics of these groups were also examined. All propensity score analyses were performed with one-to-one or many-to-one matching, depending on group size. Patients were matched based on their propensity scores with a margin of error of 0.2 standard deviations using the nearest neighbor method without replacement. The goodness of fit of propensity score analysis was evaluated by the Hosmer–Lemeshow test.

Data for continuous variables are presented as medians with interquartile ranges. Categorical data are presented as frequencies and percentages. Patient characteristics and outcomes were compared between the 2 groups using the Mann–Whitney *U* test (for numerical variables), Fisher exact test (for categorical variables), and chi-square tests (for categorical variables). All analyses were performed using R statistical software version 3.6.3 (The Institute of Statistical Mathematics, Tokyo, Japan). All reported *P* values are 2-tailed, and differences with *P* < .05 were considered statistically significant.

## 3. Results

**Figure 1.**
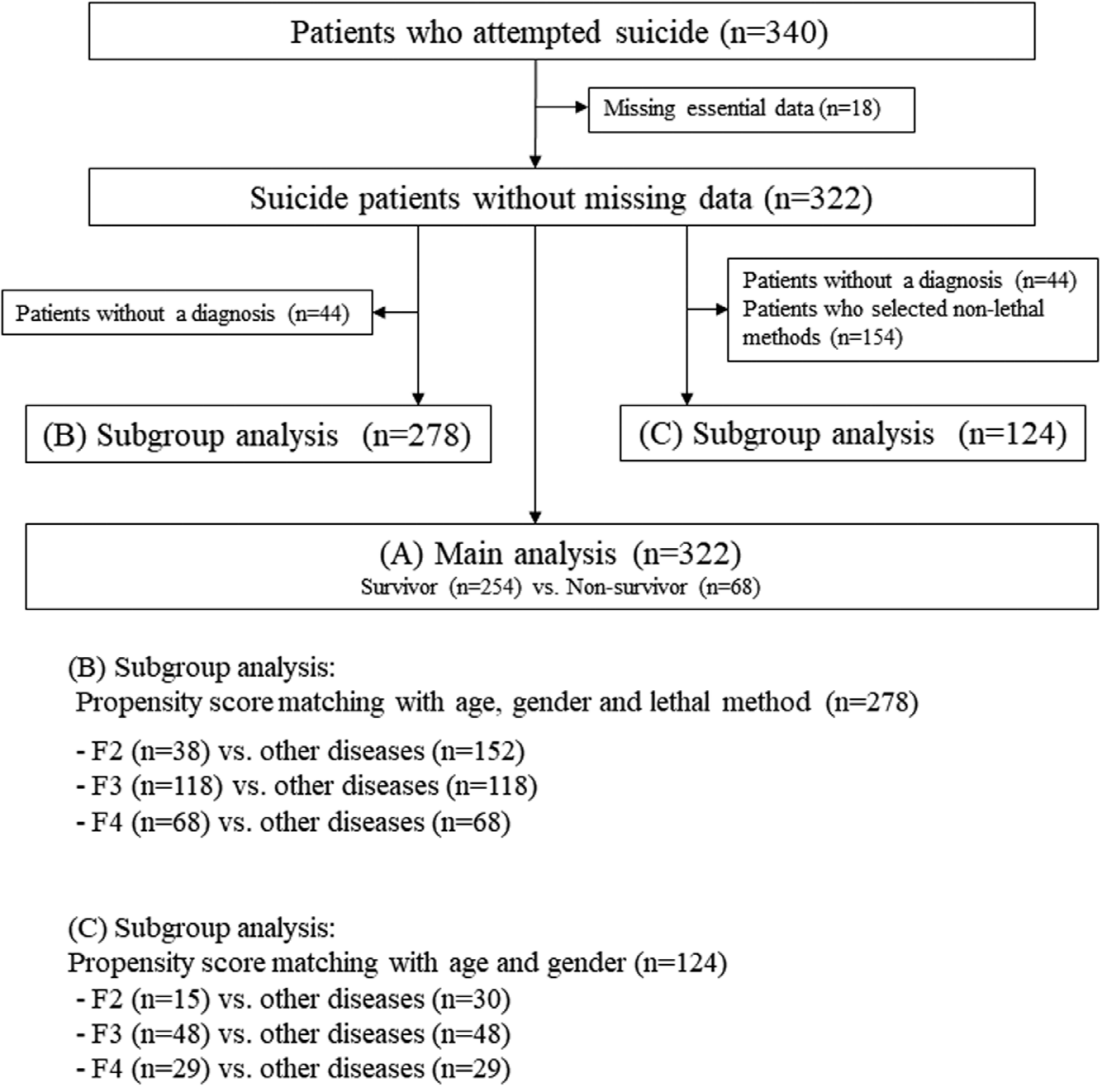
Flow chart of patient enrollment in this study.

Of the 340 patients studied, patients with missing essential data (n = 18) were excluded, and the remaining 322 patients were included in the analysis (Fig. 1). All patients excluded from the analysis owing to missing essential data were fatal cases.

Table 1 shows the variables for the 2 groups of survivors and non-survivors. In the non-survivor group, there were more older adults, men, and patients without a history of psychiatric treatment. Alternatively, in the survivor group, more patients had attempted suicide in public, and more patients had consumed alcohol shortly before the suicide. There was no significant difference between the 2 groups in the percentage of patients who had previously attempted suicide. According to the ICD-10 classification of disorders, most patients were in F2, F3, and F4. In the non-survivor group, the proportion of patients with F3 and those who had not been diagnosed was high. The most common method of suicide was hanging, followed by cutting, overdose, jumping, poisoning, CO intoxication, and burning (Table 1). The mortality rate by suicide method was high for hanging (60.6%), burning (27.3%), jumping (21.6%), and CO intoxication (8.8%), in that order. There were no overdose deaths in this study.

**Table 1:** Patients’ baseline characteristics.

Variables	Survivor (n = 254)	Non-survivor (n = 68)	*P* value
Age, yr	40 [26–55]	49 [38–61]	.005
Gender; male (n, %)	108 (42.5)	43 (63.2)	.003
Spouse; yes (n, %)	77 (30.6)	25 (41.0)	.129
Housemate; yes (n, %)	163 (64.2)	48 (76.2)	.075
Suicide attempt in public (n, %)	58 (22.8)	7 (10.3)	.026
Drinking alcohol before suicide attempt; yes (n, %)	55 (22.1)	1 (3.7)	.022
Suicide attempt witnessed by a bystander (n, %)	20 (7.9)	1 (1.5)	.092
Past suicide attempts; yes (n, %)	95 (40.6)	16 (48.5)	.452
Psychiatric history			.033
Undergoing treatment (n, %)	148 (58.7)	25 (46.3)	
Termination or suspension (n, %)	24 (9.5)	2 (3.7)	
No psychiatric history (n, %)	80 (31.7)	27 (50.0)	
ICD classification			
F0	12 (5.1)	1 (2.4)	
F1	13 (5.5)	1 (2.4)	
F2	30 (12.7)	8 (9.5)	
F3	107 (45.3)	28 (66.7)	
F4	64 (27.1)	5 (11.9)	
F5	2 (0.6)	1 (2.4)	
F6	14 (5.9)	0 (0.0)	
F7	3 (1.3)	0 (0.0)	
F8	17 (7.2)	2 (4.8)	
No diagnosis	19 (7.5)	26 (38.2)	
Duplication of psychiatric disease; yes (n, %)	27 (11.4)	4 (9.5)	1.000
Method			
Hanging	26 (10.2)	40 (58.8)	
Cutting	53 (20.9)	4 (5.9)	
Overdose	52 (20.5)	0 (0.0)	
Jumping	40 (15.7)	11 (16.2)	
Poisoning	35 (13.8)	3 (4.4)	
CO intoxication	31 (12.2)	3 (4.4)	
Burn	8 (3.1)	3 (4.4)	
Others	9 (3.5)	4 (5.9)	
Psychiatric medication			
Benzodiazepine	126 (51.6)	8 (11.8)	
Antidepressant	83 (34.0)	7 (10.3)	
Antipsychotic	78 (32.0)	3 (4.4)	
Mood stabilizer	22 (9.0)	0 (0)	
Antiepileptic	11 (4.5)	0 (0)	
Anti-dementia	3 (1.2)	0 (0)	
No medication	98 (40.2)	26 (38.2)	
No data	10 (3.9)	33 (47.9)	
APACHE II score	12.0 [7.0–18.0]	32.0 [26.0–33.0]	<.001
Unfavorable neurological outcome (n, %)	38 (15.0)	68 (100.0)	<.001

The non-survivor group included more older adults, men, and patients with no history of psychiatric treatment than the survivor group, and the survivor group had patients who attempted suicide in public and consumed alcohol before the attempt.

F0 = organic and including symptomatic and mental disorders. F1 = mental and behavioral disorders due to psychoactive substance use. F2 = schizophrenia, schizotypal and delusional disorders, F3 = mood disorders, F4 = neurotic, stress-related, and somatoform disorders, F5 = behavioral syndromes associated with physiological disturbances and physical factors, F6 = disorders of adult personality and behavior, F7 = mental retardation, F8 = disorders of psychological development.

Second, a subgroup analysis was performed to evaluate the outcomes of patients with ICD-10 classifications F2, F3, and F4 (Fig. 1). Propensity score matching was performed using age, gender, and lethal methods as covariates, which were considered to have a significant impact on the results of this study. The results for F2 versus non-F2 patients, F3 versus non-F3 patients, and F4 versus non-F4 patients are shown in Table 2. Although there was no significant statistical difference, there was a tendency for poor neurological outcome in F2 (Table 2A), high hospital mortality in F3 (Table 2B), and low in-hospital mortality in F4 (Table 2C). The detailed results of this subgroup analysis are shown in Supplemental Table 1, http://links.lww.com/MD/J740. The results of the Hosmer–Lemeshow test indicated that all *P* values were not below 0.05, and there were no problems with the goodness of fit of the propensity score matching.

**Table 2:** Subgroup analysis of patients with ICD-10 classifications F2, F3, and F4.

A)Comparison of ICD-10 classification F2 and non-F2 patients after propensity score matching			
Variables	F2 (n = 38)	non-F2 (n = 152)	*P* value
Age, yr	41.0 [31.0–52.0]	41.0 [32.0–53.0]	.810
Gender; male (n, %)	14 (36.8)	58 (38.2)	1.000
Lethal method (n, %)	15 (39.5)	72 (38.2)	1.000
Outcomes			
Unfavorable neurological outcome (n, %)	14 (36.8)	32 (21.0)	.056
In-hospital mortality (n, %)	8 (21.0)	23 (15.1)	.461
B)Comparison of ICD-10 classification F3 and non-F3 patients after propensity score matching			
Variables	F3 (n = 118)	non-F3 (n = 118)	*P* value
Age, yr	40.0 [29.3–53.0]	41.0 [29.0–55.8]	.910
Gender; male (n, %)	52 (44.1)	10 (41.5)	.793
Lethal method (n, %)	51 (43.2)	51 (43.2)	1.000
Outcomes			
Unfavorable neurological outcome (n, %)	35 (29.7)	34 (28.8)	1.000
In-hospital mortality (n, %)	24 (20.3)	13 (11.0)	.072
C)Comparison of ICD-10 classification F4 and non-F4 patients after propensity score matching			
Variables	F4 (n = 68)	non-F4 (n = 68)	*P* value
Age, yr	35.0 [23.0–50.0]	39.0 [29.0–52.0]	.320
Gender; male (n, %)	23 (33.8)	18 (26.5)	.455
Lethal method (n, %)	29 (42.6)	30 (44.1)	1.000
Outcomes			
Unfavorable neurological outcome (n, %)	14 (20.6)	20 (29.4)	.322
In-hospital mortality (n, %)	5 (7.4)	13 (19.1)	.074

F2 = schizophrenia, schizotypal and delusional disorders, F3 = mood disorders, F4 = neurotic, stress-related, and somatoform disorders.

Finally, the same analysis was performed only for the patients who selected the lethal methods (Fig. 1 results are shown in Table 3). The covariates for propensity score matching in this analysis were age and gender. Patients who selected the lethal method tended to have worse neurological outcomes for F2 and significantly worse hospital mortality for F3 (Table 3A and 3B). There was no significant difference between F4 and the outcome (Table 3C). Detailed results of the subgroup analysis in patients subjected to lethal methods are shown in Supplemental Table 2, http://links.lww.com/MD/J741.

**Table 3:** Subgroup analysis of patients with ICD-10 classifications F2, F3, and F4 who selected lethal methods.

A)Comparison of ICD-10 classification F2 and Non-F2 patients after propensity score matching			
Variables	F2 (n = 15)	non-F2 (n = 30)	*P* value
Age, yr	41.0 [33.0–53.0]	42.5 [33.3–53.0]	.809
Gender; male (n, %)	5 (33.3)	11 (36.7)	1.000
Outcomes			
Unfavorable neurological outcome (n, %)	12 (80.0)	14 (46.7)	.054
In-hospital mortality (n, %)	8 (53.3)	8 (26.7)	.105
B)Comparison of ICD-10 classification F3 and non-F3 patients after propensity score matching			
Variables	F3 (n = 48)	non-F3 (n = 48)	*P* value
Age, yr	37.0 [26.8–45.5]	36.5 [24.8–47.5]	.950
Gender; male (n, %)	20 (41.7)	18 (37.5)	.835
Outcomes			
Unfavorable neurological outcome (n, %)	31 (64.6)	23 (47.9)	.149
In-hospital mortality (n, %)	20 (41.7)	9 (18.8)	.025
C)Comparison of ICD-10 classification F4 and non-F4 patients after propensity score matching			
Variables	F4 (n = 29)	non-F4 (n = 29)	*P* value
Age, yr	29.0 [22.0–39.0]	31.0 [22.0–38.0]	.876
Gender; male (n, %)	10 (34.5)	11 (37.9)	1.000
Outcomes			
Unfavorable neurological outcome (n, %)	12 (41.4)	18 (62.1)	.189
In-hospital mortality (n, %)	4 (13.8)	11 (37.9)	.070

F2 = schizophrenia, schizotypal and delusional disorders, F3 = mood disorders, F4 = neurotic, stress-related, and somatoform disorders.

Additionally, as alcohol consumption shortly before suicide was thought to affect the outcome in this analysis, the characteristics between groups of patients divided by the presence of alcohol consumption are shown in Supplemental Table 3, http://links.lww.com/MD/J743. This analysis was performed on 276 patients with no missing data. Patients who drank alcohol shortly before suicide had favorable neurological outcomes, and this trend remained even among those who chose lethal methods.

## 4. Discussion

This study retrospectively analyzed the effect of the type of psychiatric disorder on the acute outcome of suicide. In the overall analysis, there were more older adults, men, and patients with no history of psychiatric treatment in the non-survivor group. Conversely, the survivor group showed a higher incidence of attempted suicide in public and more alcohol consumption prior to the act. Subgroup analysis revealed that patients with ICD-10 classification F2 (i.e., schizophrenia, schizotypal, and delusional disorders) had poor neurological outcomes, which was also more pronounced in patients who chose lethal methods. Patients with ICD-10 classification F3, or mood disorders, exhibited worse in-hospital mortality, with significant implications for patients who selected lethal methods.

Suicide mortality rates were higher among men and older adults, which is consistent with previous studies.^[12–14,16]^ Patients who received psychiatric interventions had lower suicide rates, aligning with previous reports.^[17]^ The methods of suicide with the highest mortality rates tended to be the same as those reported in the past.^[14,15]^

Regarding specific psychiatric disorders, outcomes for ICD-10 classifications F2 and F3 tended to be worse than that of other psychiatric disorders, while F4 showed relatively better outcomes. It has been reported that people with psychiatric disorders have a higher risk of suicide than the general population.^[18]^ However, no study has compared acute suicide mortality rates for each psychiatric disorder. This may suggest that F2 and F3 patients have a stronger desire to complete suicide and choose lethal methods, whereas F4 patients, with lower anxiety tolerance, may act impulsively without a deep obsession with death. The analysis results, even with limited methods of suicide, supported this hypothesis (Table 3). Furthermore, the results of this study revealed that F3 patients had a high rate of hospital visits but a low survival rate, whereas F4 patients tended to have a low rate of hospital visits but a high survival rate. Education of general practitioners and non-psychiatrist physicians, treatment with cognitive-behavioral therapy, and restriction of access to lethal methods are important to prevent suicide in F3 patients.^[19]^ Therefore, a multidimensional support system beyond psychiatrists is considered necessary to prevent suicide deaths in F3 patients. Additionally, care management does not significantly reduce the risk of self-harm in patients with frequent suicidal ideation. Interventions that appear to be helpful may harm some people.^[20]^ It has also been suggested that the method of intervention and the selection of the target population selection are also important in therapeutic intervention. F3 includes major depressive disorder and bipolar disorder, but it is difficult to differentiate between them.^[21]^ Among patients with bipolar disorder, 60% were first diagnosed with major depressive disorder,^[22]^ and 56% of were later diagnosed with major depressive disorder.^[23]^ As the pharmacotherapeutic strategies for major depressive disorder and bipolar disorder are quite different, it may be possible that underestimation of bipolar disorder or incorrect initial treatment strategies may have affected patient outcomes.

There is an association between alcohol consumption and increased suicide rates and mortality.^[24–26]^ However, these reports discuss the risk of long-term alcohol use for suicide. In this study, suicide attempts while drinking were associated with a lower mortality rate. Alcohol use just before the attempt was shown to be a rather favorable prognostic factor. Alcohol intoxication depletes attentional resources and reduces the drinker awareness of matters unrelated to the primary task at hand.^[27]^ Therefore, when patients drink alcohol just before committing suicide, their attention is focused solely on carrying out the suicide attempt. As a result, they may not be able to correctly assess the likelihood of death by their chosen method, which may lead to an increased survival rate. In the long term, alcohol drinkers have a higher risk of suicide, and repeated suicide attempts reduce survival.

## 5. Limitations and strengths

This study was conducted retrospectively in a single institution, and the number of patients enrolled was relatively small. Another limitation of the study is the lack of details on psychotropic medication and treatment course, and the lack of detailed scoring of psychiatric symptoms. Although this study adopts propensity score matching, there are many potential confounders and effect modifiers, such as medications, smoking, history of non-psychiatric disorders, treatment after hospitalization, and other unmeasured factors. The use of ICD-10 to classify psychiatric disorders precludes the assessment of disease-specific effects (e.g., major depressive disorder vs bipolar disorder). Although medical data are carefully described by several physicians, reporting bias may exist as not all information is described. Dead patients who were excluded from the analysis, or patients with minor injuries who were not transported to the study facility, may have caused the study results to be inaccurate (selection bias). In addition, the lack of objective indicators of alcohol use, such as blood levels and assessment of the degree of intoxication, which may be a limitation of this study. As this study was performed at a single site, the generalizability of the findings may be considered limited. It may not be possible to extrapolate the results of this study to other medical institutions, especially those located abroad or in community hospitals.

The strength of this study is that it is the first attempt to address the problem of treating suicide attempters and suicide survivors separately, unlike in previous studies. It is clear that the means of choice and prognosis for suicide differ by disease. This study is the first cross-sectional evaluation (pilot study) of suicide rates, prognoses, and means of choice by disease, while minimizing to the greatest extent possible, the inevitable biases in suicide research. The results and perspectives of this study may be used to generate future high-quality research.

## 6. Conclusion

Among all patients with suicidal tendencies, lower survival rates were observed in older people, men, and those without a history of psychiatric treatment. Notably, patients who consumed alcohol shortly before the suicide exhibited a more favorable outcome. When considering specific diseases, patients with stress-related and somatoform disorders tended to have higher survival rates, albeit with lower hospital visitation rates. Conversely, patients with mood disorders showed higher hospital visitation rates but lower survival rates, particularly among those who chose lethal methods.

## Acknowledgments

We would like to thank Editage (https://www.editage.jp/) for English language editing.

## Author contributions

**Conceptualization:** Takumi Tsuchida, Hisashi Narita.

**Data curation:** Masaki Takahashi.

**Formal analysis:** Masaki Takahashi.

**Methodology:** Takumi Tsuchida, Masaki Takahashi, Hisashi Narita, Takeshi Wada.

**Project administration:** Takumi Tsuchida, Takeshi Wada.

**Software:** Masaki Takahashi.

**Supervision:** Hisashi Narita, Takeshi Wada.

**Validation:** Asumi Mizugaki, Hisashi Narita.

**Writing – original draft:** Takumi Tsuchida.

**Writing – review & editing:** Takumi Tsuchida, Asumi Mizugaki, Takeshi Wada.

## Supplementary Material






