# Supplementary material for: Differences in acute outcomes of suicide patients by psychiatric disorder: Retrospective observational study
Source: Medicine (Baltimore). 2023 Sep 22;102(38):e35065. doi: 10.1097/MD.0000000000035065 (PMC10519571; doi:10.1097/MD.0000000000035065)
Supplement: Supplementary file 1 [file medi-102-e35065-s001.docx]

Supplemental Table 1. Details of patient background by psychiatric disorder, and results of propensity score matching by age, gender, and lethal method

1. Comparison of ICD-10 classification F2 and non-F2 patients

| Variables | before propensity score matching (n=278) | | | after propensity score matching (n=190) | | |
| --- | --- | --- | --- | --- | --- | --- |
|  | F2 (n=38) | non-F2 (n=240) | *p*-Value | F2 (n=38) | non-F2 (n=152) | *p*-Value |
| Age, yr | 41 [31-52] | 40 [27-55] | 0.748 | 41 [31-52] | 41 [32-53] | 0.810 |
| Gender; male (n, %) | 14 (36.8) | 103 (42.9) | 0.596 | 14 (36.8) | 58 (38.2) | 1.000 |
| Spouse; yes (n, %) | 12 (33.3) | 76 (32.3) | 1.000 | 12 (33.3) | 55 (36.9) | 0.847 |
| Housemate; yes (n, %) | 23 (63.9) | 160 (67.5) | 0.705 | 23 (63.9) | 103 (68.7) | 0.692 |
| Suicide attempt at public (n, %) | 9 (23.7) | 46 (19.2) | 0.514 | 9 (23.7) | 25 (16.4) | 0.344 |
| Drinking alcohol before attempt; yes (n, %) | 3 (9.7) | 44 (20.7) | 0.221 | 3 (9.7) | 29 (21.8) | 0.206 |
| Witness by bystander, n (%) | 15 (6.2) | 4 (10.5) | 0.307 | 4 (10.5) | 4 (2.6) | 0.052 |
| Past suicide attempts; yes (n, %) | 10 (34.5) | 97 (47.5) | 0.233 | 10 (34.5) | 64 (50.0) | 0.152 |
| Psychiatric history |  |  | 0.507 |  |  | 0.472 |
| Undergoing treatment (n, %) | 21 (61.8) | 150 (65.8) |  | 21 (61.8) | 101 (69.2) |  |
| Termination or suspension (n, %) | 5 (14.7) | 20 (8.8) |  | 5 (14.7) | 12 (8.2) |  |
| No psychiatric history (n, %) | 8 (23.5) | 58 (25.4) |  | 8 (23.5) | 33 (22.6) |  |
| Lethal method (n, %) | 15 (39.5) | 109 (45.4) | 0.599 | 15 (39.5) | 58 (38.2) | 1.000 |
| Method |  |  |  |  |  |  |
| Hanging | 3 (7.9) | 43 (17.9) |  | 3 (7.9) | 27 (17.8) |  |
| Overdose | 5 (13.2) | 47 (19.6) |  | 5 (13.2) | 36 (23.7) |  |
| Cut | 10 (26.3) | 43 (17.9) |  | 10 (26.3) | 28 (18.4) |  |
| Jumping | 10 (26.3) | 34 (14.2) |  | 10 (26.3) | 15 (9.9) |  |
| Poisoning | 7 (18.4) | 29 (12.1) |  | 7 (18.4) | 22 (14.5) |  |
| CO intoxication | 1 (2.6) | 25 (10.4) |  | 1 (2.6) | 13 (8.6) |  |
| Burn | 1 (2.6) | 7 (2.9) |  | 1 (2.6) | 3 (2.0) |  |
| Others | 1 (2.6) | 12 (5.0) |  | 1 (2.6) | 8 (5.3) |  |
| Psychiatric medication |  |  |  |  |  |  |
| Benzodiazepine | 16 (51.6) | 115 (56.1) |  | 16 (51.6) | 74 (58.7) |  |
| Antidepressant | 4 (12.9) | 85 (41.5) |  | 4 (12.9) | 57 (45.2) |  |
| Antipsychotic | 18 (58.1) | 62 (30.2) |  | 18 (58.1) | 43 (34.1) |  |
| Mood stabilizer | 2 (6.5) | 20 (9.8) |  | 2 (6.5) | 13 (10.3) |  |
| Antiepileptic | 1 (3.2) | 10 (4.9) |  | 1 (3.2) | 5 (4.0) |  |
| Anti-dementia | 0 (0.0) | 3 (1.5) |  | 0 (0.0) | 1 (0.8) |  |
| No medication | 13 (41.9) | 71 (34.6) |  | 13 (41.9) | 40 (31.7) |  |
| APACHE II score | 13.0 [6.5-18.5] | 12.5 [7.0-18.0] | 0.784 | 13.0 [6.5-18.5] | 12.0 [6.0-17.0] | 0.707 |
| Outcomes |  |  |  |  |  |  |
| Unfavorable neurological outcome (n, %) | 14 (36.8) | 64 (26.7) | 0.243 | 14 (36.8) | 32 (21.1) | 0.056 |
| In-hospital mortality (n, %) | 8 (21.1) | 34 (14.2) | 0.327 | 8 (21.1) | 23 (15.1) | 0.461 |

1. Comparison of ICD-10 classification F3 and non-F3 patients.

| Variables | before propensity score matching (n=278) | | | after propensity score matching (n=136) | | |
| --- | --- | --- | --- | --- | --- | --- |
|  | F3 (n=135) | non-F3 (n=143) | *p*-Value | F3 (n=118) | non-F3 (n=118) | *p*-Value |
| Age, yr | 41 [33-56] | 39 [24-53] | 0.046 | 40 [29-53] | 41 [29-56] | 0.910 |
| Gender; male (n, %) | 62 (45.9) | 55 (38.5) | 0.226 | 52 (44.1) | 49 (41.5) | 0.793 |
| Spouse; yes (n, %) | 42 (32.1) | 46 (32.9) | 0.898 | 35 (30.4) | 41 (35.3) | 0.484 |
| Housemate; yes (n, %) | 83 (62.4) | 100 (71.4) | 0.123 | 73 (62.4) | 81 (69.8) | 0.269 |
| Suicide attempt at public (n, %) | 26 (19.3) | 29 (20.3) | 0.881 | 20 (16.9) | 28 (23.7) | 0.257 |
| Drinking alcohol before attempt; yes (n, %) | 28 (24.6) | 19 (14.6) | 0.053 | 26 (25.5) | 14 (13.2) | 0.034 |
| Witness by bystander, n (%) | 7 (5.2) | 12 (8.4) | 0.346 | 6 (5.1) | 11 (9.3) | 0.314 |
| Past suicide attempts; yes (n, %) | 49 (47.1) | 58 (45.0) | 0.792 | 39 (42.9) | 43 (40.2) | 0.773 |
| Psychiatric history |  |  | <0.001 |  |  | 0.001 |
| Undergoing treatment (n, %) | 96 (77.4) | 75 (54.3) |  | 86 (78.2) | 62 (54.9) |  |
| Termination or suspension (n, %) | 9 (7.3) | 16 (11.6) |  | 8 (7.3) | 12 (10.6) |  |
| No psychiatric history (n, %) | 19 (15.3) | 47 (34.1) |  | 16 (14.5) | 39 (34.5) |  |
| Lethal method (n, %) | 67 (49.6) | 57 (39.9) | 0.117 | 51 (43.2) | 51 (43.2) | 1.000 |
| Method |  |  |  |  |  |  |
| Hanging | 28 (20.7) | 18 (12.6) |  | 23 (19.5) | 14 (11.9) |  |
| Cut | 24 (17.8) | 28 (19.6) |  | 24 (20.3) | 19 (16.1) |  |
| Overdose | 20 (14.8) | 33 (23.1) |  | 19 (16.1) | 29 (24.6) |  |
| Jumping | 13 (9.6) | 31 (21.7) |  | 12 (10.2) | 31 (26.3) |  |
| Poisoning | 16 (11.9) | 20 (14.0) |  | 16 (13.6) | 15 (12.7) |  |
| CO intoxication | 22 (16.3) | 4 (2.8) |  | 15 (12.7) | 3 (2.5) |  |
| Burn | 4 (3.0) | 4 (2.8) |  | 1 (0.8) | 3 (2.5) |  |
| Others | 8 (5.9) | 5 (3.5) |  | 8 (6.8) | 4 (3.4) |  |
| Psychiatric medication |  |  |  |  |  |  |
| Benzodiazepine | 67 (64.4) | 64 (48.5) |  | 60 (65.2) | 55 (50.5) |  |
| Antidepressant | 58 (55.8) | 31 (23.5) |  | 52 (56.5) | 29 (26.6) |  |
| Antipsychotic | 34 (32.7) | 46 (34.8) |  | 30 (32.6) | 40 (36.7) |  |
| Mood stabilizer | 9 (8.7) | 13 (9.8) |  | 8 (8.7) | 10 (9.2) |  |
| Antiepileptic | 5 (4.8) | 6 (4.5) |  | 5 (5.4) | 5 (4.6) |  |
| Anti-dementia | 0 (0.0) | 3 (2.3) |  | 0 (0.0) | 2 (1.8) |  |
| No medication | 26 (25.0) | 58 (43.9) |  | 22 (23.9) | 46 (42.2) |  |
| APACHE II score | 13.0 [8.0-20.5] | 11.5 [6.0-17.3] | 0.114 | 13.0 [8.0-21.0] | 13.0 [6.5-18.0] | 0.072 |
| Outcomes |  |  |  |  |  |  |
| Unfavorable neurological outcome (n, %) | 41 (30.4) | 37 (25.9) | 0.425 | 35 (29.7) | 34 (28.8) | 1.000 |
| In-hospital mortality (n, %) | 28 (20.7) | 14 (9.8) | 0.012 | 24 (20.3) | 13 (11.0) | 0.072 |

1. Comparison of ICD-10 classification F4 and non-F4 patients.

| Variables | before propensity score matching (n=278) | | | after propensity score matching (n=136) | | |
| --- | --- | --- | --- | --- | --- | --- |
|  | F4 (n=69) | non-F4 (n=209) | *p*-Value | F4 (n=68) | non-F4 (n=68) | *p*-Value |
| Age, yr | 33 [23-50] | 41 [30-56] | 0.017 | 35 [23-50] | 39 [29-52] | 0.320 |
| Gender; male (n, %) | 23 (33.3) | 94 (45.0) | 0.094 | 23 (33.8) | 18 (26.5) | 0.455 |
| Spouse; yes (n, %) | 24 (35.3) | 64 (31.5) | 0.654 | 24 (35.8) | 20 (30.3) | 0.581 |
| Housemate; yes (n, %) | 53 (77.9) | 130 (63.4) | 0.037 | 52 (77.6) | 48 (71.6) | 0.552 |
| Suicide attempt at public (n, %) | 11 (15.9) | 44 (21.1) | 0.389 | 11 (16.2) | 11 (16.2) | 1.000 |
| Drinking alcohol before attempt; yes (n, %) | 12 (18.5) | 35 (19.6) | 1.000 | 12 (18.8) | 13 (22.0) | 0.662 |
| Witness by bystander, n (%) | 3 (4.3) | 16 (7.7) | 0.422 | 3 (4.4) | 5 (7.4) | 0.718 |
| Past suicide attempts; yes (n, %) | 34 (52.3) | 73 (43.5) | 0.243 | 33 (51.6) | 26 (46.4) | 0.589 |
| Psychiatric history |  |  | <0.001 |  |  | <0.001 |
| Undergoing treatment (n, %) | 34 (49.3) | 137 (71.0) |  | 34 (50.0) | 50 (79.4) |  |
| Termination or suspension (n, %) | 5 (7.2) | 20 (10.4) |  | 5 (7.4) | 7 (11.1) |  |
| No psychiatric history (n, %) | 30 (43.5) | 36 (18.7) |  | 29 (42.6) | 6 (9.5) |  |
| Lethal method (n, %) |  |  |  | 15 (40.5) | 72 (38.9) | 0.856 |
| Method |  |  |  |  |  |  |
| Hanging | 11 (15.9) | 35 (16.7) |  | 11 (16.2) | 14 (20.6) |  |
| Overdose | 17 (24.6) | 35 (16.7) |  | 16 (23.5) | 14 (20.6) |  |
| Cut | 14 (20.3) | 39 (18.7) |  | 14 (20.6) | 11 (16.2) |  |
| Jumping | 15 (21.7) | 29 (13.9) |  | 15 (22.1) | 9 (13.2) |  |
| Poisoning | 8 (11.6) | 28 (13.4) |  | 8 (11.8) | 11 (16.2) |  |
| CO intoxication | 3 (4.3) | 23 (11.0) |  | 3 (4.4) | 5 (7.4) |  |
| Burn | 0 (0.0) | 8 (3.8) |  | 0 (0.0) | 2 (2.9) |  |
| Others | 1 (1.4) | 12 (5.7) |  | 1 (1.5) | 2 (2.9) |  |
| Psychiatric medication |  |  |  |  |  |  |
| Benzodiazepine | 33 (50.0) | 98 (57.6) |  | 33 (50.8) | 35 (66.0) |  |
| Antidepressant | 17 (25.8) | 72 (42.4) |  | 17 (26.2) | 24 (45.3) |  |
| Antipsychotic | 11 (16.7) | 69 (40.6) |  | 11 (16.9) | 27 (50.9) |  |
| Mood stabilizer | 5 (7.6) | 17 (10.0) |  | 5 (7.7) | 7 (13.2) |  |
| Antiepileptic | 2 (3.0) | 9 (5.3) |  | 2 (3.1) | 6 (11.3) |  |
| Anti-dementia | 0 (0.0) | 3 (1.8) |  | 0 (0.0) | 0 (0.0) |  |
| No medication | 30 (45.5) | 54 (31.8) |  | 29 (44.6) | 10 (18.9) |  |
| APACHE II score | 10.0 [5.5-17.5] | 13.0 [7.0-19.0] | 0.118 | 10.5 [5.3-17.8] | 11.0 [7.0-20.5] | 0.228 |
| Outcomes |  |  |  |  |  |  |
| Unfavorable neurological outcome (n, %) | 14 (20.3) | 64 (30.6) | 0.122 | 14 (20.6) | 20 (29.4) | 0.322 |
| In-hospital mortality (n, %) | 5 (7.2) | 37 (17.7) | 0.034 | 5 (7.4) | 13 (19.1) | 0.074 |

Data presented as median [25th-75th percentile] or numbers (%).

F2: schizophrenia, schizotypal and delusional disorders, F3: mood disorders, F4: neurotic, stress-related and somatoform disorders.

The APACHE II score was calculated only for hospitalized patients, and not for patients who died in the Emergency Department.

The unfavorable neurological outcome is defined by the cerebral performance category (CPC) scale 3 to 5 at 28-hospital day.
