# Supplementary material for: Differences in acute outcomes of suicide patients by psychiatric disorder: Retrospective observational study
Source: Medicine (Baltimore). 2023 Sep 22;102(38):e35065. doi: 10.1097/MD.0000000000035065 (PMC10519571; doi:10.1097/MD.0000000000035065)
Supplement: Supplementary file 3 [file medi-102-e35065-s003.docx]

Supplemental Table 3. Differences in patient background according to drinking just before suicide.

| Variables | All patients (n=276) | | | Patients who select the lethal method (n=126) | | |
| --- | --- | --- | --- | --- | --- | --- |
|  | Drinking (n=56) | No drinking (n=220) | *p*-Value | Drinking (n=21) | No drinking (n=105) | *p*-Value |
| Age, yr | 41 [31-52] | 40 [26-56] | 0.775 | 41 [33-46] | 38 [24-52] | 0.417 |
| Gender; male (n, %) | 25 (44.6) | 96 (43.6) | 1.000 | 11 (52.4) | 50 (47.6) | 0.812 |
| Spouse; yes (n, %) | 17 (30.9) | 70 (32.1) | 1.000 | 6 (30.0) | 29 (27.9) | 1.000 |
| Housemate; yes (n, %) | 30 (53.6) | 153 (69.5) | 0.027 | 10 (47.6) | 79 (75.2) | 0.017 |
| Suicide attempt at public (n, %) | 39 (69.6) | 179 (81.4) | 0.066 | 7 (33.3) | 24 (22.9) | 0.404 |
| Witness by bystander, n (%) | 3 (5.4) | 17 (7.7) | 0.774 | 1 (4.8) | 8 (7.6) | 1.000 |
| Past suicide attempts; yes (n, %) | 17 (33.3) | 85 (41.7) | 0.338 | 7 (35.0) | 44 (44.9) | 0.466 |
| Psychiatric history |  |  | 0.162 |  |  | 0.017 |
| Undergoing treatment (n, %) | 28 (50.0) | 126 (57.8) |  | 6 (28.6) | 59 (56.7) |  |
| Termination or suspension (n, %) | 9 (16.1) | 17 (7.8) |  | 5 (23.8) | 7 (6.7) |  |
| No psychiatric history (n, %) | 19 (33.9) | 75 (34.4) |  | 10 (47.6) | 38 (36.5) |  |
| Lethal method (n, %) | 21 (37.5) | 105 (47.7) | 0.180 | 21 (100.0) | 105 (100.0) | NA |
| ICD classification |  |  |  |  |  |  |
| F0 | 0 (0.0) | 13 (6.6) |  | 0 (0.0) | 3 (3.5) |  |
| F1 | 6 (12.8) | 6 (3.0) |  | 4 (28.6) | 2 (2.4) |  |
| F2 | 3 (6.4) | 28 (14.2) |  | 0 (0.0) | 9 (10.6) |  |
| F3 | 28 (59.6) | 86 (43.7) |  | 7 (50.0) | 44 (51.8) |  |
| F4 | 12 (25.5) | 53 (26.9) |  | 5 (35.7) | 21 (24.7) |  |
| F5 | 1 (2.1) | 1 (0.5) |  | 0 (0.0) | 0 (0.0) |  |
| F6 | 0 (0.0) | 14 (7.1) |  | 0 (0.0) | 6 (7.1) |  |
| F7 | 0 (0.0) | 3 (1.5) |  | 0 (0.0) | 3 (3.5) |  |
| F8 | 4 (8.5) | 13 (6.6) |  | 0 (0.0) | 7 (8.2) |  |
| Method |  |  | NA |  |  | 0.206 |
| Hanging | 4 (7.1) | 37 (16.8) |  | 4 (19.0) | 37 (35.2) |  |
| Overdose | 15 (26.8) | 35 (15.9) |  |  |  |  |
| Cut | 10 (17.9) | 44 (20.0) |  |  |  |  |
| Jumping | 7 (12.5) | 35 (15.9) |  | 7 (33.3) | 35 (33.3) |  |
| Poisoning | 5 (8.9) | 31 (14.1) |  |  |  |  |
| CO intoxication | 9 (16.1) | 23 (10.5) |  | 9 (42.9) | 23 (21.9) |  |
| Burn | 1 (1.8) | 10 (4.5) |  | 1 (4.8) | 10 (9.5) |  |
| Others | 5 (8.9) | 5 (2.3) |  |  |  |  |
| Psychiatric medication |  |  |  |  |  |  |
| Benzodiazepine | 24 (43.6) | 104 (50.5) |  | 7 (33.3) | 45 (46.9) |  |
| Antidepressant | 20 (36.4) | 64 (31.1) |  | 7 (33.3) | 32 (33.3) |  |
| Antipsychotic | 15 (27.3) | 63 (30.6) |  | 4 (19.0) | 27 (28.1) |  |
| Mood stabilizer | 7 (12.7) | 15 (7.3) |  | 1 (4.8) | 6 (6.2) |  |
| Antiepileptic | 2 (3.6) | 9 (4.4) |  | 0 (0.0) | 3 (3.1) |  |
| Anti-dementia | 0 (0.0) | 3 (1.5) |  | 0 (0.0) | 2 (2.1) |  |
| No medication | 27 (49.1) | 86 (41.7) |  | 14 (66.7) | 43 (44.8) |  |
| APACHE II score | 10.0 [6.0-21.0] | 12.0 [7.0-18.0] | 0.608 | 11.0 [6.0-21.0] | 12.0 [6.0-20.5] | 0.863 |
| Outcomes |  |  |  |  |  |  |
| Unfavorable neurological outcome (n, %) | 5 (8.9) | 58 (26.4) | 0.004 | 4 (19.0) | 48 (45.7) | 0.029 |
| In-hospital mortality (n, %) | 1 (1.8) | 26 (11.8) | 0.022 | 1 (4.8) | 22 (21.0) | 0.120 |

Data presented as median [25th-75th percentile] or numbers (%).

F0: organic, including symptomatic, mental disorders, F1: mental and behavioral disorders due to psychoactive substance use, F2: schizophrenia, schizotypal and delusional disorders, F3: mood disorders, F4: neurotic, stress-related and somatoform disorders, F5: behavioral syndromes associated with physiological disturbances and physical factors, F6: disorders of adult personality and behavior, F7: mental retardation, F8: disorders of psychological development.

The APACHE II score was calculated only for hospitalized patients, and not for patients who died in the Emergency Department.

The unfavorable neurological outcome is defined by the cerebral performance category (CPC) scale 3 to 5 at 28-hospital day.
